# Transcription factor SS18L1 regulates the proliferation, migration and differentiation of Schwann cells in peripheral nerve injury

**DOI:** 10.3389/fvets.2022.936620

**Published:** 2022-08-15

**Authors:** Tianmei Qian, Pingping Qiao, Yingnan Lu, Hongkui Wang

**Affiliations:** ^1^Suzhou Medical College of Soochow University, Suzhou, China; ^2^Key Laboratory of Neuroregeneration of Jiangsu and Ministry of Education, Co-Innovation Center of Neuroregeneration, NMPA Key Laboratory for Research and Evaluation of Tissue Engineering Technology Products, Nantong University, Nantong, China; ^3^School of Overseas Education, Changzhou University, Changzhou, China

**Keywords:** transcription factor, SS18L1, Schwann cell, proliferation, migration, differentiation, peripheral nerve injury

## Abstract

Transcription factors bind to specific DNA sequences, modulate the transcription of target genes, and regulate various biological processes, including peripheral nerve regeneration. Our previous analysis showed that SS18L1, a gene encoding the transcription factor SS18-like protein 1, was differentially expressed in the distal sciatic nerve stumps after rat sciatic nerve transection injury, but its effect on peripheral nerve injury has not been reported. In the current study, we isolated and cultured primary Schwann cells, and examined the role of SS18L1 for the biological functions of the cells. Depletion of SS18L1 by siRNA in Schwann cells enhanced cell proliferation and inhibited cell migration, as determined by EdU assay and transwell migration assay, respectively. In addition, silencing of SS18L1 inhibited Schwann cell differentiation induced by HRG and cAMP. Bioinformatics analyses revealed an interaction network of SS18L1, including DF2, SMARCD1, SMARCA4, and SMARCE1, which may be implicated in the regulatory functions of SS18L1 on the proliferation, migration and differentiation of Schwann cells. In conclusion, our results revealed a temporal expression profile of SS18L1 in peripheral nerve injury and its potential roles during the process of nerve recovery.

## Introduction

A transcription factor is a sequence-specific DNA binding protein that regulates the transcription rates of target genes (1, 2). To date, ~120,000 transcription factors of 73 transcription factor families and 80,000 transcription cofactors of 83 transcription cofactor families have been identified in 97 animal genomes (3). The human genome encodes more than 2,000 transcription factors (4). These transcription factors act as master switches, turn on or off the expressions of numerous target genes, and regulate multiple physiological and pathological processes (4, 5). Therefore, transcription factor-based therapies show great potential in the clinical treatment of many diseases (6–9).

Peripheral nerve injury is an important clinical problem that can be induced by a variety of causes, including accidental trauma, physical injury, pathological damage, and degenerative diseases (10, 11). Various transcription factors, including c-Jun, activating transcription factor 3 (ATF3), cAMP response element binding protein (CREB), signal transducer and activator of transcription-3 (STAT3), CCAAT/enhancer binding proteins β and δ (C/EBPs), Oct-6, Sox11, p53, nuclear factor kappa-light-chain-enhancer of activated B cell (NF-κB), and ELS-like 3 (ELK3), have been recognized as key regulators of the biological activities of neurons and Schwann cells and potentially essential players in peripheral nerve regeneration (10). Our previous study demonstrated that genes encoding for many other transcription factors, such as FOS-like antigen 1 (FOSL1), B-cell CLL/lymphoma 11A zinc finger protein (BCL11A), SMAD family member 3 (SMAD3), CASK interacting protein 1 (CASKIN1), and SS18-like protein 1 (SS18L1) were differentially expressed in distal sciatic nerve stumps after sciatic nerve transection in rats, implying that these transcription factors may also be critical in regulating the phenotypes of Schwann cells in peripheral nerve stumps during peripheral nerve regeneration (12).

SS18L1 gene encodes the calcium-responsive transactivator (CREST) protein, which is a chromatin remodeling protein. The SS18L1 protein consists of three functional domains, an N-terminal auto-regulatory domain that suppresses transactivation in the basal state, a central methionine-rich domain involved in protein-protein interaction, and a C-terminal glutamine-rich domain responsible for transactivation (13). Northern blot, *in situ* hybridization, immunohistochemistry and Western blotting analysis all showed that the expression of SS18L1 is enriched in the brain (14). SS18L1 is an essential component of the nBAF neuron-specific chromatin remodeling complex that is related to the chromatin remodeling complex called switch/sucrose non-fermentable (SWI/SNF) (15). It plays an important role for the normal development of the nervous system, such as neuronal growth and differentiation (16). SS18L1 is involved in cell cycle regulation and differentiation of neurons through switching SWI/SNF by replacing its homologous protein SS18 in the complex (14). In addition, SS18L1 is shown to bind chromatin remodeling proteins BAF250 and BRG-1, and plays a crucial role in the differentiation and maturation of the spermatogenic epithelial cells (14). Furthermore, SS18L1 can interact with histone acetyltransferases p300 and CREB-binding protein (CBP), thus regulating the determination and differentiation of testicular tissues as well as the metabolic remodeling in the later stages of spermatogenesis (14, 17). We have previously shown that the expression of SS18L1 is significantly down-regulated in rat distal sciatic nerve stumps at early time points after peripheral nerve injury (12), but its role in the peripheral nerve system remains unclear. In the current study, we investigated the effect of SS18L1 silencing on key cellular processes in Schwann cells during peripheral nerve regeneration, including proliferation, migration and differentiation.

## Materials and methods

### Animal surgery

Adult and neonatal Sprague-Dawley (SD) rats were purchased from the Laboratory Animal Center of Nantong University. All experimental procedures were ethically approved by the Institutional Animal Care and Use Committee of Laboratory Animals center of Nantong University (Inspection No: S20210105-013). A total of 30 6- to 8-week adult male SD rats, weighing 180-200 g were randomly separated into 5 groups (at 0, 1, 4, 7, and 14 days after nerve injury) at 6 rats per group, and used for sciatic nerve crush as previously described (18). Briefly, SD rats were anesthetized by an intraperitoneal injection of compound anesthetic (chloral hydrate 4.25 g, magnesium sulfate 2.12 g, sodium pentobarbital 886 mg, ethanol 14.25 ml, and propylene glycol 33.8 ml in 100 ml) at a dose of 0.2–0.3 mL/100 g body weight. After anaesthetization, the left sciatic nerves of rats were exposed, a 3 mm long nerve was crushed three times for 10 s each time with a 3 s interval using hemostatic forceps. After the surgical incisions were closed, the rats were housed in large cages with corncob padding in a temperature- and humidity-controlled environment (room temperature 23 ± 2°C, relative humidity 55 ± 5%) with a 12 h light/dark cycle and were allowed free access to water and food. Under anesthesia with compound anesthetic, 5 mm-long sciatic nerve segments at the crushed site together with both nerve ends, were collected at 0, 1, 4, 7, and 14 days after surgery, and then the animals were immediately euthanized by cervical dislocation.

### Real-time RT-PCR

Total RNA was isolated from cultured Schwann cells using RNA-Quick Purification Kit (Yishan Biotechnology Co., LTD, Shanghai, China) and then reversely transcribed into cDNA using HiScript^®^ III RT SuperMix for qPCR (+gDNA wiper) (Vazyme, Nanjing, Jiangsu, China). Briefly, 1 μg of RNA sample and 4 μl of 4 × gDNA wiper Mix were incubated at 42°C for 3 min, then, 4 μl of 5 × HiScript III qRT SuperMix was added and the reaction mixture was incubated at 37°C for 15 min and 85°C for 5 s. The cDNA was subjected to real-time PCR analysis of gene expression using SYBR Green Premix Ex Taq (TaKaRa) on a StepOne Real-time PCR machine (Applied Biosystems, Foster City, CA, USA). The thermocycling program was as follows: initial denaturation at 95°C for 5 min; 40 cycles of denaturation at 95°C for 30 s, annealing at 60°C for 45 s and extension at 72°C for 30 s; final extension at 72°C for 5 min. The relative gene expression level was calculated using the 2^−Δ*ΔCt*^ method with GAPDH as the reference gene. Primers of rat SS18L1, P0, MBP, or GAPDH were designed by National Center for Biotechnology Information Primer-BLAST specific primer designing tool and synthesized by Sangon Biotech (Sangon, Shanghai, China). The sequences of primers were as follows: SS18L1 (NCBI accession Nos. NM_138918) (forward) 5′-TGCAGAGCCCATGAGTCAAC-3′ and (reverse) 5′-GTCGTAGCTCTGCTCTGTGT-3′; P0 (NCBI accession Nos. NM_001314068) (forward) 5′-CGTGATCGGTGGCATCCTC-3′ and (reverse) 5′-GGCATACAGCACTGGCGTCT-3′; MBP (NCBI accession Nos. NM_001025289) (forward) 5′-CCGACGAGCTTCAGACCATC-3′ and (reverse) 5′-AGTACTTGGATCCGTGTCGC3′; and GAPDH (Accession Nos. NM_017008) (forward) 5′-AACGACCCCTTCATTGAC-3′ and (reverse) 5′-TCCACGACATACTCAGCAC-3′. The experiment was repeated three times.

### Immunostaining

Collected sciatic nerves were fixed with 4% paraformaldehyde, dehydrated with 30% sucrose, embedded in optimal cutting temperature (OCT) compound, and cut into 12 μm tissue sections using a cryostat. Nerve sections were blocked with Immunol Staining Blocking Buffer (Beyotime, Shanghai, China) for 30 min, and incubated with primary antibodies mouse anti-S100β antibody (1:300 dilution, Sigma, S2532, St. Louis, MO, USA) and rabbit anti-SS18L1 antibody (1:300 dilution, Proteintech, 12439-1-AP, Rosemont, IL, USA), rabbit anti-P0 antibody (1:100 dilution, Abcam, ab183868, Cambridge, MA, USA) or rabbit anti-MBP antibody (1:300 dilution, Abcam, ab218011) overnight at 4°C followed by incubation with secondary antibodies goat anti-mouse IgG-Alex-488 (1:1000 dilution, Abcam, ab150117) and goat anti-rabbit IgG-Cy3 (1:1000 dilution, Abcam, ab6939) at room temperature for 2 h. Nuclear staining was performed using DAPI Fluoromount-G^®^ (SouthernBiotech, 0100-20, Birmingham, AL, USA). Immunostaining images were obtained under a fluorescence microscopy (Axio Imager M2, Carl Zeiss Microscopy GmbH, Jena, Germany). The fluorescence intensity were conducted by calculations of the crushed areas using Image J software (https://imagej.nih.gov/ij/index.html). The experiment was repeated three times.

### Isolation and culture of primary Schwann cells

A total of 60 1-day-old neonatal SD rats were used to isolate Schwann cells as previously described (19). Briefly, the rat was first anesthetized by freezing on ice and decapitated with a large scissor, and then the body was sterilized with a 70% ethanol spray. The gluteus and hamstrings on the upper dorsal thigh were carefully separated to expose the underlying sciatic nerve. Subsequently, the nerve was gently separated from the surrounding muscle and membranous tissue, and an incision was conducted at the knee to isolate the nerve. The isolated sciatic nerves were digested with 3 mg/mL collagenase (Sigma) at 37°C for 30 min, followed by 0.25% trypsin (Sigma) digestion at 37°C for 10 min. The dissociated Schwann cells were cultured in dishes with Dulbecco's modified Eagle's medium (DMEM; Invitrogen, Carlsbad, CA) containing 10% fetal bovine serum (FBS; Gibco, Grand Island, NY, USA) and 1% penicillin and streptomycin (Invitrogen, Carlsbad, CA, USA) overnight in a humidified 5% CO_2_ incubator at 37°C. The dishes were pre-coated with poly-L-lysine (Sigma). Then the medium was replaced with an equal volume of DMEM/10% FBS supplemented with 10 μM Ara-C (Sigma) for additional 2 days to eliminate fibroblasts. After removing debris by gently rinsing three times with PBS, the cells were maintained in DMEM/10% FBS supplemented with 10 ng/ml human heregulin-β1 (HRG; R&D Systems Inc., Minneapolis, MN, USA) and 2 μM forskolin (Sigma) for 3 days and then purified with anti-Thy1.1 antibody (Sigma) and rabbit complement (Sigma) to remove residual fibroblasts. The purity of Schwann cells was assessed by S100β immunostaining and the cells were used for experimental analysis when the purity reached 95%.

### Schwann cell transfection

Cultured primary Schwann cells were transfected with 3 siRNAs against SS18L1 (SS18L1-siRNA-1: CCAGAGCAAGGGCAAGACA, SS18L1-siRNA-2: CCATAGCAGATTCCAACCA, and SS18L1-siRNA-3: CAACCCAGAACATGAACCT) or a non-targeting negative control (NC-siRNA: GGCTCTAGAAAAGCCTATGC) (RiboBio, Guangzhou, Guangdong, China) for 48 h using Lipofectamine RNAiMAX reagent (Invitrogen, Thermo Fisher Scientific, Inc.) according to the manufacturer's instruction. The gene silencing efficiency was determined by real-time RT-PCR analysis. The experiment was repeated three times.

### Cell proliferation assay

A total of 2 ×10^4^ primary Schwann cells were suspended in 100 μl cultured medium and seeded onto 96-well plates pre-coated with poly-L-lysine at a density of 2 ×10^5^ cells/ml. The cells were incubated for additional 12 h after adding 50 μM EdU (RiboBio), and then fixed in 4% paraformaldehyde (Xilong Scientific, Guangzhou, China) in PBS for 30 min. The proliferation of Schwann cells was determined using the Cell-Light^TM^ EdU DNA Cell Proliferation Kit (Ribobio) according to the manufacturer's protocol. The numbers of EdU-positive cells and total cells were determined by Apollo 567 fluorescent dyes and Hoechst 33342 staining. The proliferation rate of Schwann cells was calculated by dividing the numbers of EdU-positive cells by the numbers of total cells. Images were taken using a DMR fluorescence microscope (Leica Microsystems, Bensheim, Germany). The experiment was repeated three times. The three non-overlapping fields in each well were used for statistics.

### Cell migration assay

A total of 4 ×10^4^ primary Schwann cells were suspended in 100 μl DMEM medium and seeded onto the upper chamber of a 6.5 mm transwell chamber with 8 μm pores (Costar, Cambridge, MA, USA) at a density of 4 ×10^5^ cells/ml. Schwann cells were incubated for additional 24 h after filling the bottom chamber with 500 μl culture medium. The bottom of the transwell chamber was pre-coated with 10 μg/ml fibronectin (EMD Millipore Corporation, Temecula, CA, USA). After cleaning cells left on the upper surface, the transwell chambers were stained with 0.1% crystal violet (Beyotime) for 20 min at room temperature. Images were taken using a DMR inverted microscope (Leica Microsystems) and the relative migration ability of Schwann cells was calculated by measuring crystal violet-stained areas in randomly selected fields. The areas of migratory Schwann cells were calculated using Image J software. The experiment was repeated three times.

### Cell differentiation

Schwann cell differentiation was induced by treatment of HRG and cAMP as previously described (20). Briefly, Schwann cell were cultured in DMEM/F12 containing 0.5% FBS and 1% penicillin and streptomycin, and treated with 20 ng/ml HRG and 1 mM db-cAMP (Sigma) for 72 h. Then the cells were harvested for RNA extraction and subsequent real-time RT-PCR analysis. The experiment was repeated three times.

### Bioinformatics analysis

SS18L1-centered genetic network was constructed using the ClueGo (v2.5.8) and CluePedia (v1.5.8) plug-ins of the Cytoscape software (v3.8.2). CluePedia was used to identify the potential target genes of SS18L1. Gene ontology (GO, v13.05.2021) and Kyoto Encyclopedia of Genes and Genomes (KEGG, v13.05.2021) by GluoGO were used to enrich GO categories and KEGG pathways of SS18L1 and potential target genes, with a P <0.05 were selected and listed. An interaction network of SS18L1, potential target genes, and involved GO categories and KEGG pathways were constructed using the Cytoscape software. Protein-protein interaction network was constructed using STRING database (https://string-db.org/). Gene expression heatmap was generated using MeV software v4.9.0 (http://www.tm4.org/). Potential regulatory network of SS18L1 was predicted using the Ingenuity Pathways Knowledge Base (IPKB) of the Ingenuity pathway analysis (IPA) software (v01-20-04, http://www.ingenuity.com/, Ingenuity Systems Inc., Redwood City, CA, USA).

### Statistical analysis

Statistical analysis was conducted with GraphPad Prism 6.0 (GraphPad Software, Inc., San Diego, CA, USA). One-way ANOVA followed by *post hoc* Dunnett's multiple comparisons test was used when comparing multiple groups, and unpaired Student's *t*-test was used to compare two groups. Results were presented as mean ± SEM and significantly differences (*P* < 0.05) were indicated by asterisks.

## Results

### Expression of SS18L1 in rat sciatic nerve stumps after peripheral nerve injury

**Figure 1:**
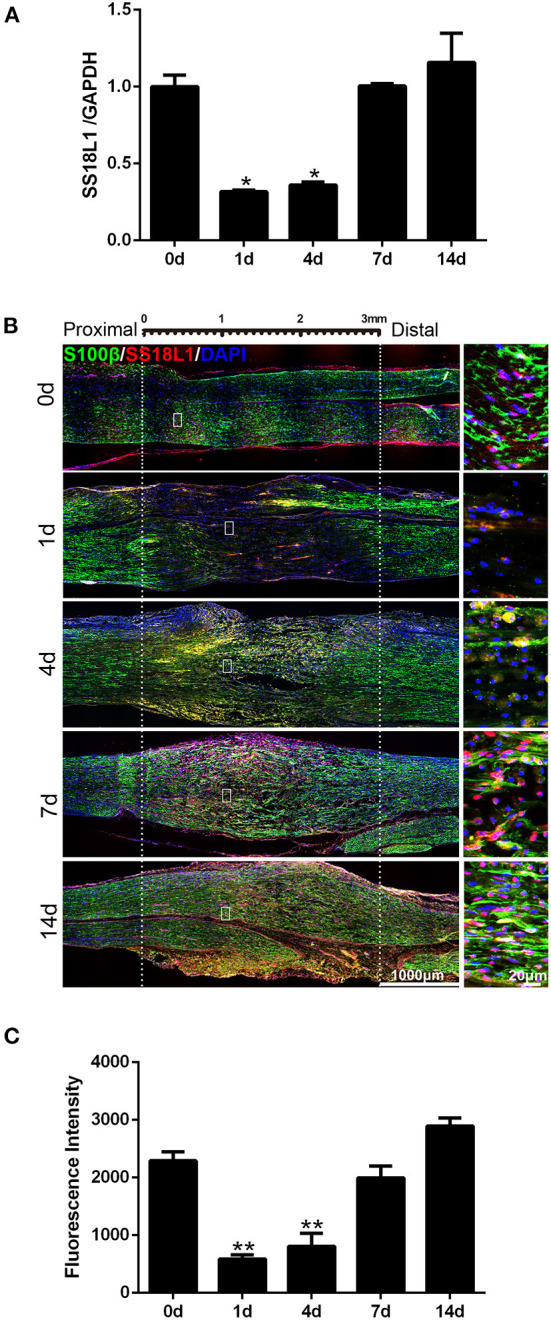
Expression of SS18L1 in rat sciatic nerve stumps after peripheral nerve injury. **(A)** The relative mRNA expression of SS18L1 at 0, 1, 4, 7, and 14 days after sciatic nerve crush injury. *P < 0.05, vs. 0 day. **(B)** Immunostaining of SS18L1 (in red) and S100β (in green) at 0, 1, 4, 7, and 14 days after sciatic nerve crush injury. DAPI (in blue) was used to stain nuclei. Scale bars indicated 1,000 μm (main image), 20 μm (magnification). **(C)** Quantification of the fluorescence intensity of SS18L1 staining in rat sciatic nerves at 0, 1, 4, 7, and 14 days after nerve injury. **P < 0.01, vs. 0 day.

Previous analyses revealed the down-regulation of SS18L1 mRNA in rat distal sciatic nerve stumps after sciatic nerve transection (12). Here, real-time RT-PCR results showed that the mRNA expression of SS18L1 rapidly decreased in the proximal sciatic nerve stumps of rats at 1 and 4 days after sciatic nerve crush, and then increased (Figure 1A). Then, immunohistochemistry was performed to determine the localization and abundance of SS18L1 protein in the rat sciatic nerve stump. The analysis demonstrated that SS18L1 protein was localized to the S100β-positive Schwann cells (Figure 1B). Strong SS18L1 expression was detected in the sciatic nerve in the absence of injury treatment (0 day). In contrast, nerve injury resulted in significantly reduced immunostaining intensity for SS18L1 at all tested time points (1, 4, 7, and 14 days), especially at the injured sites. Interestingly, SS18L1 protein levels significantly decreased 1 day after injury and then steadily increased (Figure 1B). These data indicated nerve injury could lead to suppression of SS18L1 expression at both the mRNA and protein levels, especially 1 day after the treatment (Figure 1C).

### SS18L1 is present in Schwann cells

**Figure 2:**
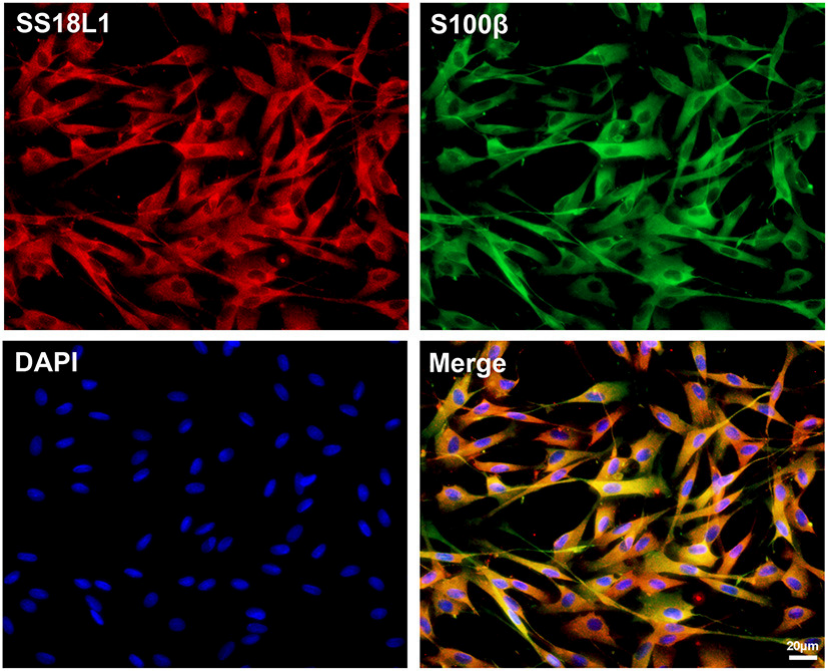
Localization of SS18L1 in cultured Schwann cells. Red color indicates SS18L1, green color indicates S100β, and blue color indicates nucleus. Scale bars indicated 20 μm.

Immunohistochemistry analysis indicated a predominant expression of SS18L1 in Schwann cells. To confirm this observation, we isolated and cultured primary Schwann cells and then detected the expression of SS18L1 by immunofluorescence staining. The purity of the cells was first verified by co-staining with the Schwann cell marker S100β and DAPI, and the result showed that more than 95% of the cells were Schwann cells. Further immunostaining of cultured Schwann cells with SS18L1 showed that the majority of SS18L1 staining overlapped with S100β staining, confirming that SS18L1 protein was expressed in Schwann cells (Figure 2).

### Inhibition of SS18L1 expression promotes Schwann cell proliferation

**Figure 3:**
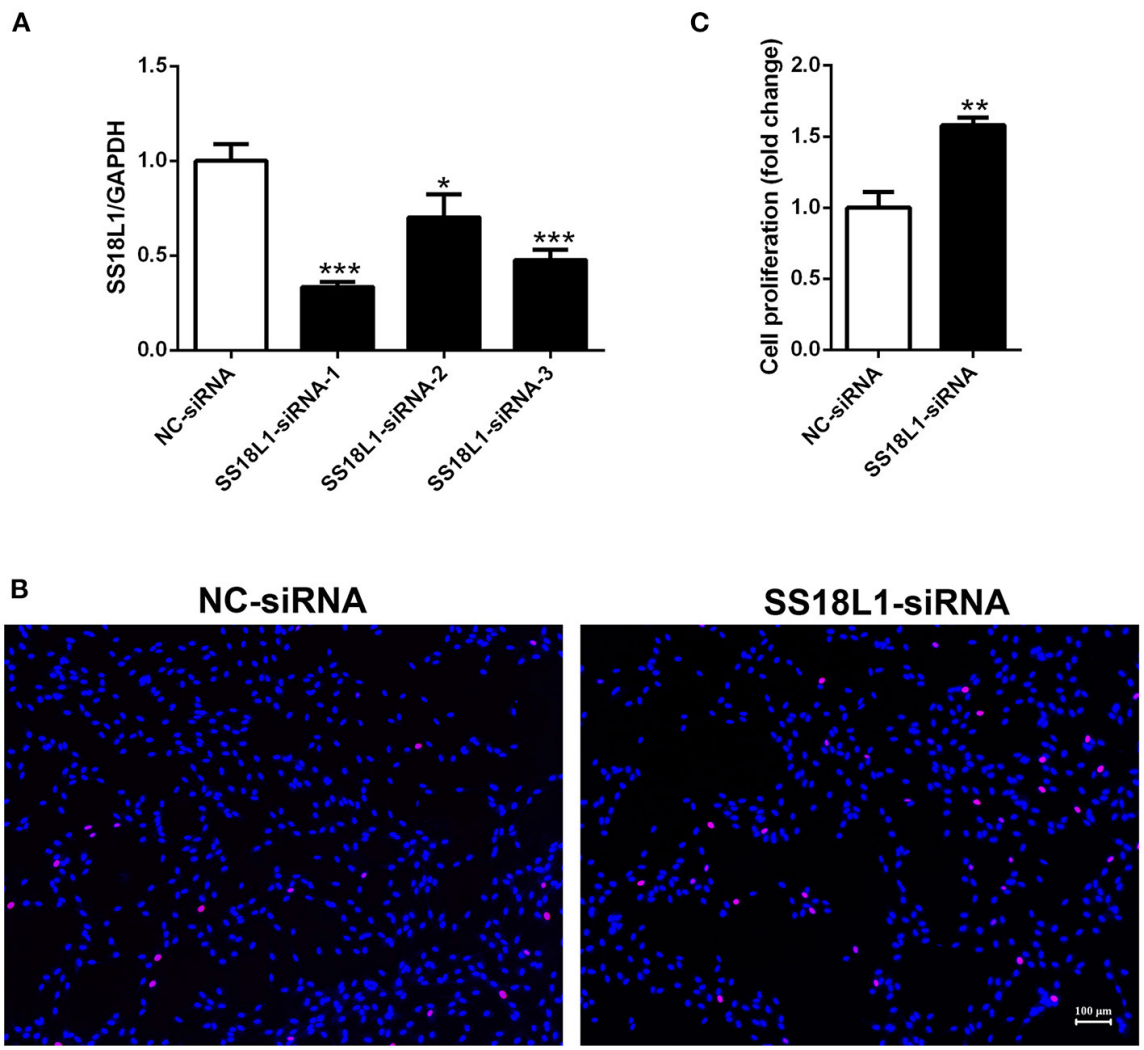
The effect of SS18L1 on Schwann cell proliferation. **(A)** Relative mRNA expression of SS18L1 in Schwann cells transfected with SS18L1 siRNAs or siRNA control (NC-siRNA). *P < 0.05, vs. siRNA control, ***P < 0.001, vs. siRNA control. **(B)** Representative images and histogram of the proliferation of Schwann cells transfected with SS18L1 siRNA or siRNA control. Red color indicates Schwann cells stained with EdU and blue color indicates Schwann cells stained with Hoechst 33342. Scale bars indicated 100 μm. **(C)** Summarized histogram of the proliferation of Schwann cells transfected with SS18L1 siRNA or siRNA control. **P < 0.01, vs. siRNA control.

The proliferation of Schwann cells after peripheral nerve injury are critical for subsequent peripheral nerve regeneration. To investigate the biological roles of SS18L1 in Schwann cell proliferation and peripheral nerve regeneration, we first transfected the cultured Schwann cells with three different siRNAs against SS18L1 or a siRNA control. RT-PCR analysis showed all the SS18L1 siRNAs could effectively inhibit SS18L1 mRNA expression (Figure 3A), of which the SS18L1-siRNA-1 was the most effective one and used in subsequent experiments. Next, we used an EdU proliferation assay to determine the role of SS18L1 in Schwann cell proliferation. The result showed the portion of proliferating cells was significantly increased in Schwann cells transfected with SS18L1 siRNA, compared to the siRNA control (Figures 3B,C), suggesting that in Schwann cells, the expression of SS18L1 has a suppressive role in regulating proliferation.

### Inhibition of SS18L1 expression reduces Schwann cell migration

**Figure 4:**
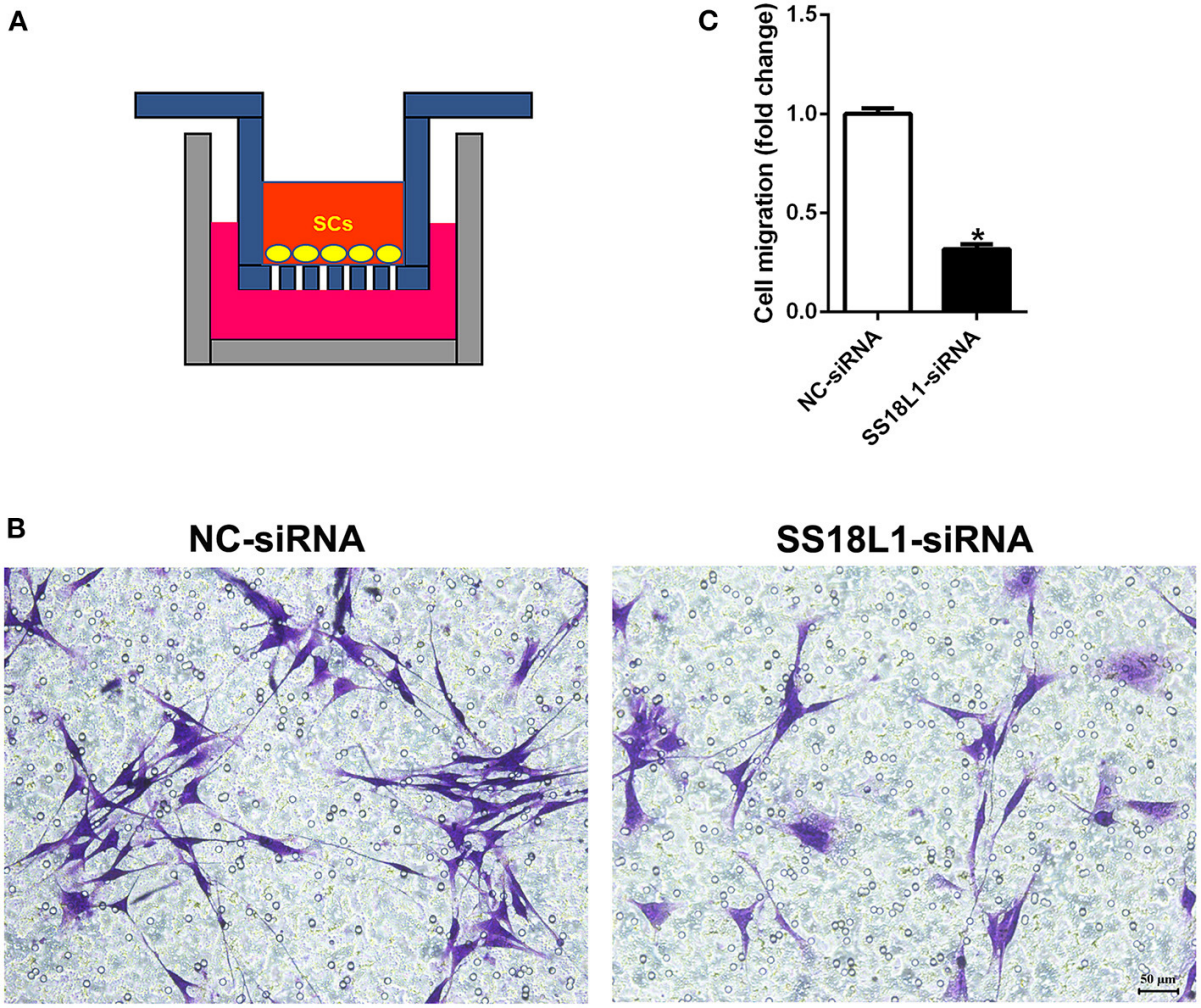
The effect of SS18L1 on Schwann cell migration. **(A)** The schematic diagram of cell migration assay. **(B)** Representative images of the migration of Schwann cells transfected with SS18L1 siRNA or siRNA control. Violet color indicated Schwann cells migrated toward the bottom surface. Scale bars indicated 50 μm. **(C)** Summarized histogram of the migration of Schwann cells transfected with SS18L1 siRNA or siRNA control. *P < 0.05, vs. siRNA control.

To assess whether SS18L1 has any effect on cell migration, we transfected Schwann cells with SS18L1 siRNA or siRNA control and performed transwell migration assays (Figure 4A). Migrated Schwann cells were stained with crystal violet in violet color (Figure 4B). The analysis showed that the SS18L1 siRNA significantly inhibited the number of migrated Schwann cells in comparison to the control (Figure 4C), indicating a pro-migratory role of SS18L1 in Schwann cells.

### Inhibition of SS18L1 expression prevents Schwann cell differentiation

**Figure 5:**
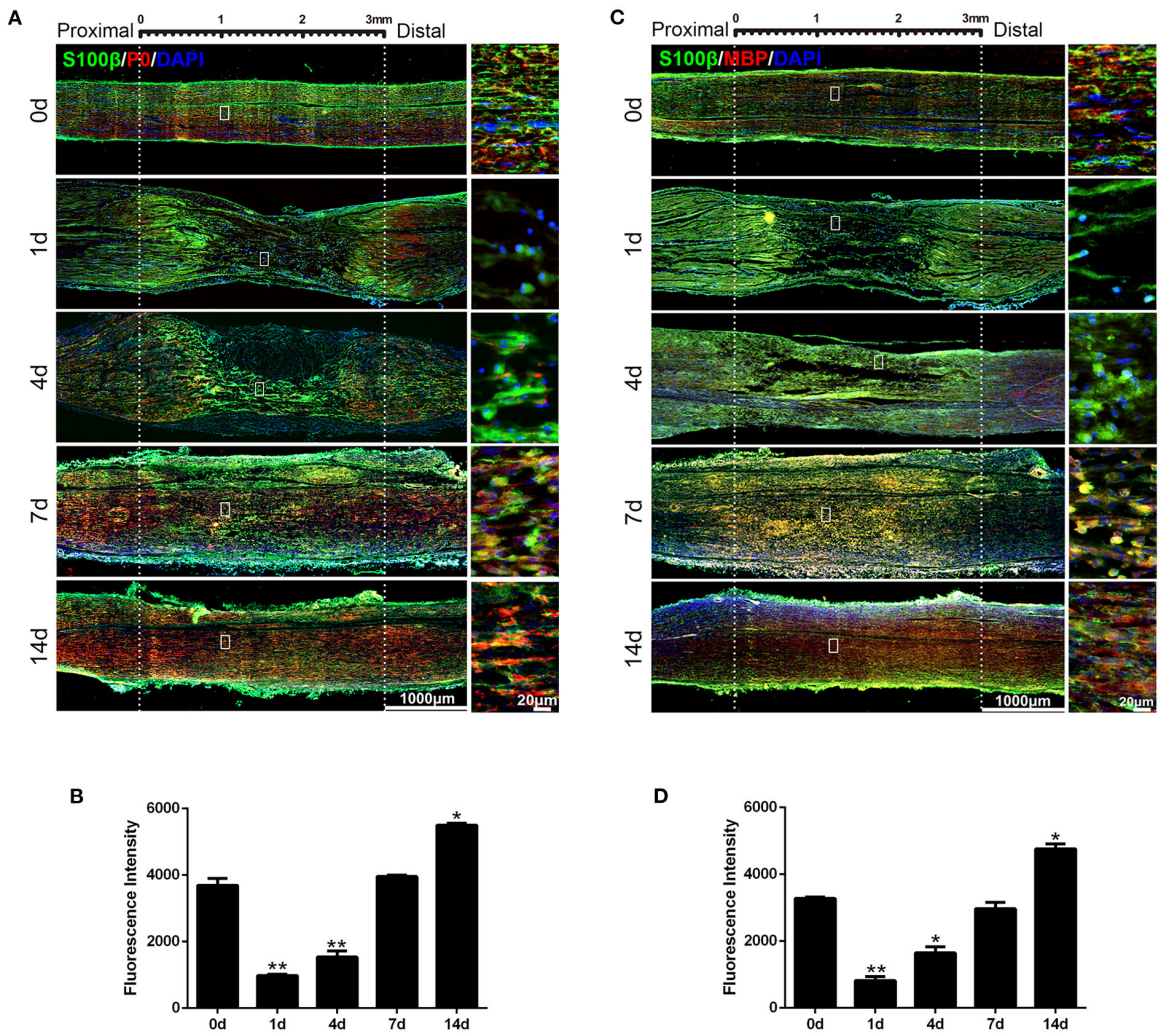
Expression of P0 and MBP in rat sciatic nerve stumps after peripheral nerve injury. **(A)** Immunostaining of P0 protein (in red) and S100β (in green) at 0, 1, 4, 7, and 14 days after sciatic nerve crush injury. DAPI (in blue) was used to label nuclei. Scale bars indicated 1,000 μm (main image), 20 μm (magnification). **(B)** Quantification of the fluorescence intensity of P0 in rat sciatic nerves at 0, 1, 4, 7, and 14 days after nerve injury. *P < 0.05, vs. 0 day, **P < 0.01, vs. 0 day. **(C)** Immunostaining of MBP protein (in red) and S100β (in green) at 0, 1, 4, 7, and 14 days after sciatic nerve crush injury. DAPI (in blue) was used to label nuclei. Scale bars indicated 1,000 μm (main image), 20 μm (magnification). **(D)** Quantification of the fluorescence intensity of MBP in rat sciatic nerves at 0, 1, 4, 7, and 14 days after nerve injury. *P < 0.05, vs. 0 day, **P < 0.01, vs. 0 day.

**Figure 6:**
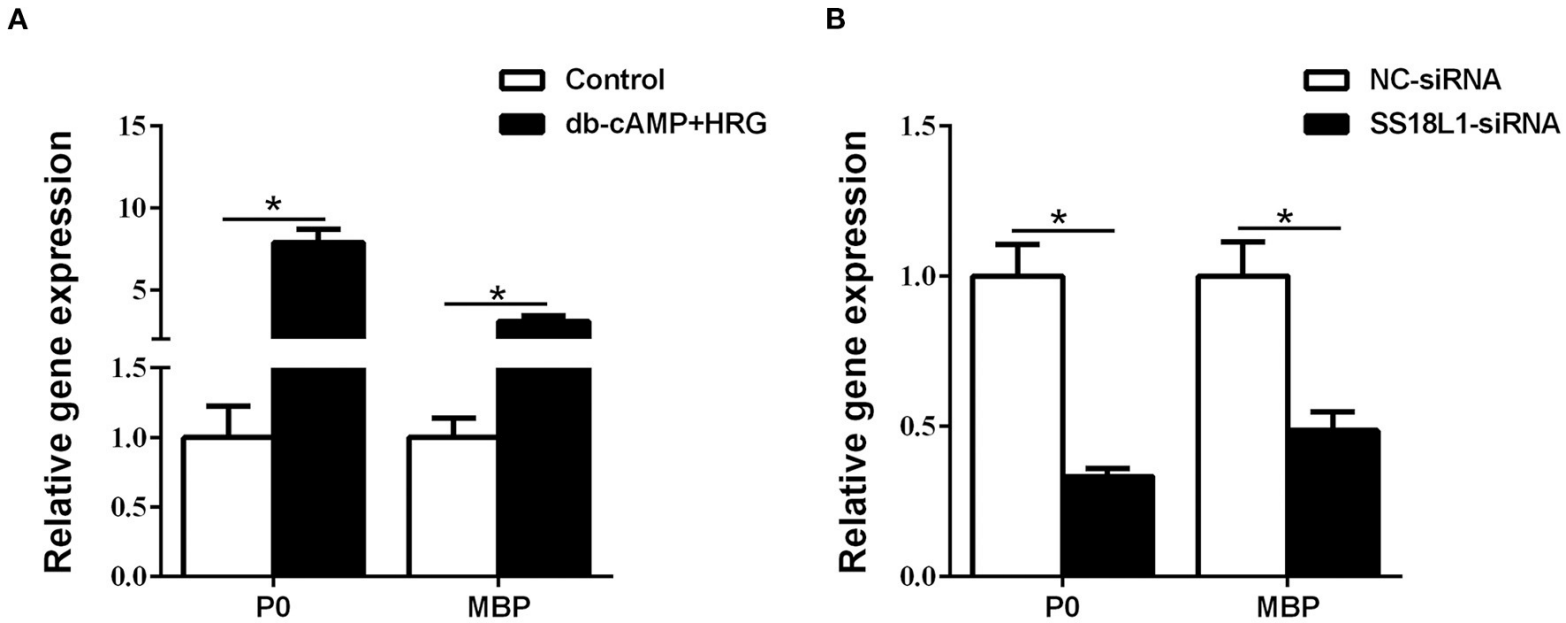
The effect of SS18L1 on Schwann cell differentiation. **(A)** The mRNA levels of P0 and MBP were higher in Schwann cells cultured in differentiation culture medium (db-cAMP + HRG) than those cultured in control medium. **(B)** The mRNA levels of P0 and MBP were higher in siRNA control transfected Schwann cells cultured in differentiation culture medium than SS18L1 siRNA transfected Schwann cells cultured in differentiation culture medium. *P < 0.05, vs. siRNA control.

The expressions of the myelin-related genes P0 (also known as MPZ) and MBP are two of the well-defined markers of matured Schwann cells. Immunohistochemistry analysis showed nerve crush significantly decreased the expression of both P0 (Figures 5A,B) and MBP (Figures 5C,D) in the sciatic nerve on the first day after injury and increased steadily thereafter, which was consistent with the expression pattern of SS18L1. These results suggested that SS18L1 might facilitate myelination by influencing Schwann cell differentiation post nerve injury. Next, we sought to verify this hypothesis by determining the effect of SS18L1 depletion on the differentiation of Schwann cells. To this end, we first set up the condition for Schwann cell differentiation. Real-time RT-PCR confirmed that treatment of HRG and cAMP effectively induced Schwann cell differentiation indicated by significant induction of P0 and MBP expression compared with the control (Figure 6A). Subsequently, Schwann cells transfected with SS18L1 siRNA or siRNA control were subjected to induction of differentiation. As shown in Figure 6B, compared with the siRNA control, SS18L1 siRNA treatment significantly inhibited P0 and MBP expression in Schwann cells, suggesting a role of SS18L1 in promoting Schwann cell differentiation.

### Identification of potential networks of SS18L1

**Figure 7:**
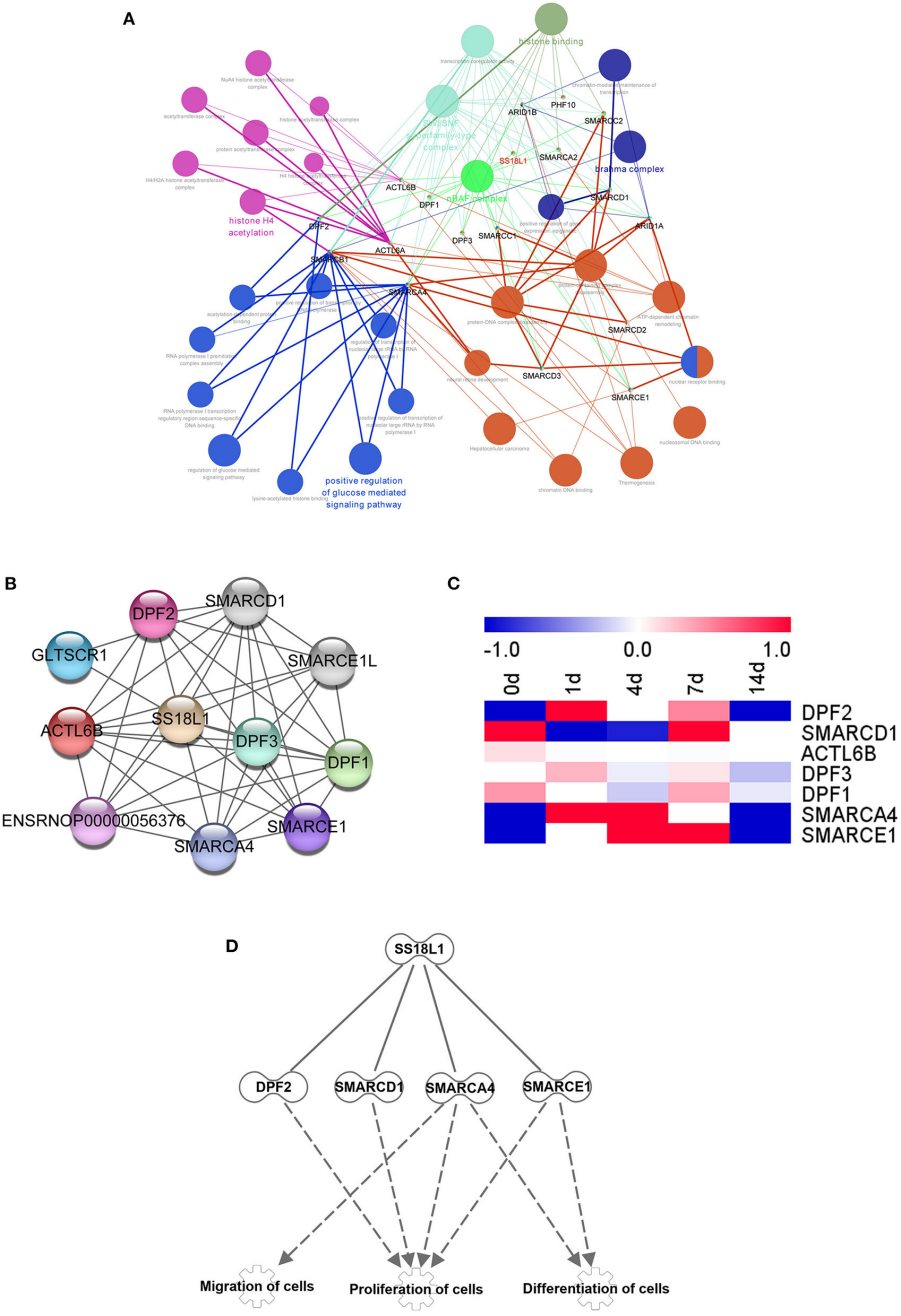
Bioinformatics analysis showing SS18L1-centered genetic network. **(A)** Cytoscape software predicted the associated genes and biological functions of SS18L1. Different colors of network nodes, respectively, represent various biological functions, while node sizes reflect the enrichment of biological functions. **(B)** STRING was used to analyze the interaction network among the selected differentially expressed genes. **(C)** The heatmap of the expression patterns of interacted genes in sciatic nerve stumps after nerve injury. Red color indicates up-regulation and blue color indicates down-regulation. **(D)** The interaction network of SS18L1 and associated proteins DF2, SMARCD1, SMARCA4, SMARCE1.

To evaluate underlying biological functions regulated by SS18L1 in Schwann cell proliferation, migration and differentiation, we used the ClueGo tool of Cytoscape software to explore potential targets of SS18L1. The analysis showed that a total of 17 genes, including DPF1 (Double PHD Fingers 1), ARID1B (AT-Rich Interaction Domain 1B), PHF10 (PHD Finger Protein 10), SMARCC2 (SWI/SNF Related, Matrix Associated, Actin Dependent Regulator Of Chromatin Subfamily C Member 2), SMARCA2 (SWI/SNF Related, Matrix Associated, Actin Dependent Regulator Of Chromatin, Subfamily A, Member 2), ACTL6B (Actin Like 6B), DPF3 (Double PHD Fingers 3), SMARCC1 (SWI/SNF Related, Matrix Associated, Actin Dependent Regulator Of Chromatin Subfamily C Member 1), SMARCD1 (SWI/SNF Related, Matrix Associated, Actin Dependent Regulator Of Chromatin, Subfamily D, Member 1), ARID1A (AT-Rich Interaction Domain 1A), ACTL6A (Actin Like 6A), DPF2 (Double PHD Fingers 2), SMARCB1 (SWI/SNF Related, Matrix Associated, Actin Dependent Regulator Of Chromatin, Subfamily B, Member 1), SMARCA4 (SWI/SNF Related, Matrix Associated, Actin Dependent Regulator Of Chromatin, Subfamily A, Member 4), SMARCD3 (SWI/SNF Related, Matrix Associated, Actin Dependent Regulator Of Chromatin, Subfamily D, Member 3), SMARCE1 (SWI/SNF Related, Matrix Associated, Actin Dependent Regulator Of Chromatin, Subfamily E, Member 1) and SMARCD2 (SWI/SNF Related, Matrix Associated, Actin Dependent Regulator Of Chromatin, Subfamily D, Member 2) could be potential targets of SS18L1. The analysis indicated that these genes are associated with several biological processes including nBAF complex, SWI/SNF superfamily-type complex, histone H4 acetylation, brahma complex and positive regulation of glucose mediated signaling pathway (Figure 7A). In addition, the protein-protein interaction networks of SS18L1 were predicted using STRING (Figure 7B). Then, the temporal expression patterns of these related genes shared by the two analysis methods in the sciatic nerve stumps after rat sciatic nerve injury were determined based on previous sequencing data and presented in a heatmap (Figure 7C). IPA bioinformatic software analyses further revealed that SS18L1 might inhibit Schwann cell proliferation via interacting with DF2, SMARCD1, SMARCA4, and SMARCE1, and promote Schwann cell migration and differentiation via interactions with SMARCA4, SMARCE1 (Figure 7D).

## Discussion

Schwann cells are unique glial cells in the peripheral nervous system and play important roles during peripheral nerve repair and regeneration (21, 22). Peripheral nerve injury evokes the transformation of Schwann cells to a newly reconfigured repair phenotype, as repair Schwann cells, which create a permissive environment for the injured axons to regenerate (23–25). Mature Schwann cells first transdifferentiate into to a repair Schwann cells and proliferate to a larger cell population after peripheral nerve injury. An increased number of Schwann cells migrate toward the injury site to engulf axon and myelin debris and to construct a regeneration path named band of Büngner, The repair Schwann cells further differentiate to a myelinating state to wrap around regenerated axons (21, 26, 27). The remarkable plasticity of Schwann cells and the status switch of Schwann cells between transdifferentiation, proliferation, migration, redifferentiation, and remyelination largely contribute to the successful regeneration and reinnervation of injured peripheral nerves.

To recognize essential factors for the plasticity of Schwann cells during peripheral nerve repair and injury, our laboratory has constructed a rat sciatic nerve injury model and determined gene expression patterns in the sciatic nerve stumps after rat sciatic nerve injury (18, 28, 29). Schwann cells respond to nerve injury by cellular reprogramming that generates a cell phenotype specialized to promote repair. There repair cells activate a sequence of supportive functions that clear redundant myelin, attract macrophages, prevent neuronal death, help axon growth and guide axons back to their targets (27, 30). Many transcription factors have been identified to be associated with this repair program. For example, the transcription factor c-Jun plays a key role in the Schwann cell injury response. After injury, c-Jun is rapidly up-regulated in Schwann cell, and includes increase in trophic support for neurons, acceleration of myelin breakdown by autophagy, promotion of Schwann cell elongation, and formation of the bands of Büngner (30–32). Our previous study revealed that many key transcription factors were differentially expressed in distal sciatic nerve stumps after rat sciatic nerve injury, including SS18L1 (12). However, the specific biological functions of these differentially expressed transcription factors have not been fully elucidated.

The mutation of the SS18L1 gene is associated with amyotrophic lateral sclerosis (ALS) (33, 34). The functional role of SS18L1 in peripheral nervous system diseases is largely unknown. In the present study, we demonstrated that SS18L1 is expressed in Schwann cells and is involved in the biological modulation of Schwann cells, including inhibiting proliferation and promoting migration and differentiation.

Schwann cells have two time-dependent phenotypes during peripheral nerve injury (20). In the early stage after peripheral nerve injury, Schwann cells react to the injury, undergo an identity change to form a repair-promoting phenotype, and then proliferate, while the repair Schwann cells undergo migration and differentiation in the later stage. Therefore, the genes regulating Schwann cell transdifferentiation or differentiation have dynamic and temporal expression patterns over the period of peripheral nerve injury. We found that SS18L1 expression was down-regulated immediately after injury and then raised steadily (Figure 1A). The inhibition of SS18L1 expression at the early stage after peripheral nerve injury might benefit Schwann cell proliferation while the up-regulation of SS18L1 at later stages might facilitate Schwann cell migration and differentiation. Immunohistochemistry staining showed that the expression pattern of SS18L1 in rat sciatic nerve stumps after peripheral nerve injury was consistent with that of the myelin-related genes P0 and MBP, indicating that SS18L1 may associate with peripheral myelination. Indeed, we further found that depletion of SS18L1 significantly inhibited Schwann cell differentiation *in vitro*. The exact function of SS18L1 in the differentiation remains unknown. It is possible that SS18L1 facilitates debris removal and creates a favorable microenvironment for peripheral nerve regeneration.

Besides the functional assessment of SS18L1, we also predicted potential genetic and protein networks of SS18L1 that may regulate Schwann cell proliferation, migration and differentiation. It has been shown that SS18L1 can physically interact with DF2, SMARCD1, SMARCA4, and SMARCE1 (35). These SS18L1-associated proteins are important regulators of cell proliferation, migration and differentiation. For example, DPF2 is required for anchorage-independent growth of H1299 cells (36), SMARCD1 is critical for the proliferation of SYO-1 cells (37) and SMARCE1 supports T to Th1 differentiation of mouse lymphocytes (38). The absence of SMARCA4 (also known as BRG1) could prematurely stagnate Schwann cell differentiation (39), and inhibit the proliferation, migration and invasion of glioma cells (40). The effects of SS18L1 on the biology of Schwann cells may involve the interaction with these proteins, which warrants further investigation.

Taken together, through morphological, cellular and molecular experiments, we explored the expression pattern of the transcription factor SS18L1 and its effects on Schwann cell proliferation, migration and differentiation after peripheral nerve injury in rats. Furthermore, the bioinformatics analysis predicted potential targets and interaction networks of SS18L1 that may be implicated in the cellular processes of Schwann cells during peripheral nerve repair. Our findings combined with more comparative medicine researches will provide new insights into the biological roles of SS18L1 in peripheral nerve regeneration and provide a molecular pathological diagnostic model, as well as a potential therapeutic target for peripheral nerve repair in clinic.

## Data availability statement

The original contributions presented in the study are included in the article/supplementary material, further inquiries can be directed to the corresponding author/s.

## Ethics statement

The animal study was reviewed and approved by the Institutional Animal Care and Use Committee of Laboratory Animals center of Nantong University.

## Author contributions

TQ and HW conceived and designed the experiments. TQ, PQ, and YL performed the experiments and contributed reagents/materials/analysis tools. TQ and PQ analyzed the data. HW wrote the manuscript. All authors contributed to the article and approved the submitted version.

## Funding

This work was supported by grants from the National Natural Science Foundation of China (Grant No. 31730031), the Natural Science Foundation of Jiangsu Province (Grant No. BK20202013), the Priority Academic Program Development of Jiangsu Higher Education Institutions of China (PAPD), and Jiangsu Provincial Double-Innovation Doctor Program (grant no. JSSCBS20211095).

## Conflict of interest

The authors declare that the research was conducted in the absence of any commercial or financial relationships that could be construed as a potential conflict of interest.

## Publisher's note

All claims expressed in this article are solely those of the authors and do not necessarily represent those of their affiliated organizations, or those of the publisher, the editors and the reviewers. Any product that may be evaluated in this article, or claim that may be made by its manufacturer, is not guaranteed or endorsed by the publisher.

## References

[1] Latchman DS. Transcription factors: an overview. Int J Biochem Cell Biol. (1997) 29(12):1305–12. 10.1016/s1357-2725(97)00085-x 9570129

[2] Lee TI Young RA. Transcription of eukaryotic protein-coding genes. Annu Rev Genet. (2000) 34:77–137. 10.1146/annurev.genet.34.1.77 11092823

[3] Hu H Miao YR Jia LH Yu QY Zhang Q Guo AY. Animaltfdb 30: a comprehensive resource for annotation and prediction of animal transcription factors. Nucleic Acids Res. (2019) 47:D33–8. 10.1093/nar/gky822 30204897 PMC6323978

[4] Brivanlou AH Darnell JE Jr. Signal transduction and the control of gene expression. Science. (2002) 295(5556):813–8. 10.1126/science.1066355 11823631

[5] Lambert SA Jolma A Campitelli LF Das PK Yin Y Albu M. The human transcription factors. Cell. (2018) 172(4):650–65. 10.1016/j.cell.2018.01.029 29425488 PMC12908702

[6] Lambert M Jambon S Depauw S David-Cordonnier MH. Targeting transcription factors for cancer treatment. Molecules. (2018) 23:1479. 10.3390/molecules23061479 29921764 PMC6100431

[7] Takei H Kobayashi SS. Targeting transcription factors in acute myeloid leukemia. Int J Hematol. (2019) 109(1):28–34. 10.1007/s12185-018-2488-1 29956082

[8] Papavassiliou KA Papavassiliou AG. Transcription factor drug targets. J Cell Biochem. (2016) 117(12):2693–6. 10.1002/jcb.25605 27191703

[9] Hishikawa A Hayashi K Itoh H. Transcription factors as therapeutic targets in chronic kidney disease. Molecules. (2018) 23:1123. 10.3390/molecules23051123 29747407 PMC6100497

[10] Patodia S Raivich G. Role of transcription factors in peripheral nerve regeneration. Front Mol Neurosci. (2012) 5:8. 10.3389/fnmol.2012.00008 22363260 PMC3277281

[11] Faroni A Mobasseri SA Kingham PJ Reid AJ. Peripheral nerve regeneration: experimental strategies and future perspectives. Adv Drug Deliv Rev. (2015) 82–83:160–7. 10.1016/j.addr.2014.11.010 25446133

[12] Yi S Tang X Yu J Liu J Ding F Gu X. Microarray and Qpcr analyses of Wallerian degeneration in rat sciatic nerves. Front Cell Neurosci. (2017) 11:22. 10.3389/fncel.2017.00022 28239339 PMC5301003

[13] Aizawa H Hu SC Bobb K Balakrishnan K Ince G Gurevich I. Dendrite development regulated by crest, a calcium-regulated transcriptional activator. Science. (2004) 303(5655):197–202. Epub 2004/01/13. 10.1126/science.1089845 14716005

[14] Du A Li L Jiao Z Zhu G Peng T Li H. Protein expression pattern of calcium-responsive transactivator in early postnatal and adult testes. Histochem Cell Biol. (2021) 155(4):491–502. 10.1007/s00418-020-01942-1 33398438 PMC8062385

[15] Park S Park SK Watanabe N Hashimoto T Iwatsubo T Shelkovnikova TA. Calcium-responsive transactivator (crest) toxicity is rescued by loss of Pbp1/Atxn2 function in a novel yeast proteinopathy model and in transgenic flies. PLoS Genet. (2019) 15:e1008308. 10.1371/journal.pgen.1008308 31390360 PMC6699716

[16] Kukharsky MS Quintiero A Matsumoto T Matsukawa K An H Hashimoto T. Calcium-responsive transactivator (crest) protein shares a set of structural and functional traits with other proteins associated with amyotrophic lateral sclerosis. Mol Neurodegener. (2015) 10:20. 10.1186/s13024-015-0014-y 25888396 PMC4428507

[17] Pradhan A Liu Y. The calcium-responsive transactivator recruits creb binding protein to nuclear bodies. Neurosci Lett. (2004) 370:191–19104ci Let1016/j.neulet.2004.08.062 15488321 10.1016/j.neulet.2004.08.062

[18] Yi S Zhang H Gong L Wu J Zha G Zhou S. Deep sequencing and bioinformatic analysis of lesioned sciatic nerves after crush injury. PLoS ONE. (2015) 10:e0143491. 10.1371/journal.pone.0143491 26629691 PMC4668002

[19] Weinstein DE Wu R. Isolation and purification of primary Schwann cells. Curr Protoc Neurosci. (2001) Chapter 3:Unit 3 17. 10.1002/0471142301.ns0317s08 18428466

[20] Yi S Liu Q Wang X Qian T Wang H Zha G. Tau modulates Schwann cell proliferation, migration and differentiation following peripheral nerve injury. J Cell Sci. (2019) 132:jcs222059. 10.1242/jcs.222059 30782778

[21] Ko PY Yang CC Kuo YL Su FC Hsu TI Tu YK. Schwann-cell autophagy, functional recovery, and scar reduction after peripheral nerve repair. J Mol Neurosci. (2018) 64(4):601–10. 10.1007/s12031-018-1056-8 29644600

[22] Xia B Gao J Li S Huang L Zhu L Ma T. Mechanical stimulation of schwann cells promote peripheral nerve regeneration via extracellular vesicle-mediated transfer of microrna 23b-3p. Theranostics. (2020) 10(20):8974–95. 10.7150/thno.44912 32802175 PMC7415818

[23] Weiss T Taschner-Mandl S Bileck A Slany A Kromp F Rifatbegovic F. Proteomics and transcriptomics of peripheral nerve tissue and cells unravel new aspects of the human schwann cell repair phenotype. Glia. (2016) 64(12):2133–53. 10.1002/glia.23045 27545331

[24] Lv W Deng B Duan W Li Y Liu Y Li Z. Schwann cell plasticity is regulated by a weakened intrinsic antioxidant defense system in acute peripheral nerve injury. Neuroscience. (2018) 382:1–13. 10.1016/j.neuroscience.2018.04.018 29684504

[25] Fornaro M Marcus D Rattin J Goral J. Dynamic environmental physical cues activate mechanosensitive responses in the repair Schwann cell phenotype. Cells. (2021) 10:425. 10.3390/cells10020425 33671410 PMC7922665

[26] Glenn TD Talbot WS. Signals regulating myelination in peripheral nerves and the Schwann cell response to injury. Curr Opin Neurobiol. (2013) 23(6):1041–8. 10.1016/j.conb.2013.06.010 23896313 PMC3830599

[27] Jessen KR Mirsky R. The success and failure of the schwann cell response to nerve injury. Front Cell Neurosci. (2019) 13:33. 10.3389/fncel.2019.00033 30804758 PMC6378273

[28] Yu J Gu X Yi S. Ingenuity pathway analysis of gene expression profiles in distal nerve stump following nerve injury: insights into wallerian degeneration. Front Cell Neurosci. (2016) 10:274. 10.3389/fncel.2016.00274 27999531 PMC5138191

[29] Zhao L Yi S. Transcriptional landscape of alternative splicing during peripheral nerve injury. J Cell Physiol. (2019) 234(5):6876–85. 10.1002/jcp.27446 30362529

[30] Jessen KR Arthur-Farraj P. Repair Schwann cell update: adaptive reprogramming, Emt, and stemness in regenerating nerves. Glia. (2019) 67(3):421–37. 10.1002/glia.23532 30632639

[31] Arthur-Farraj PJ Latouche M Wilton DK Quintes S Chabrol E Banerjee A. C-Jun reprograms Schwann cells of injured nerves to generate a repair cell essential for regeneration. Neuron. (2012) 75(4):633–47. 10.1016/j.neuron.2012.06.021 22920255 PMC3657176

[32] Jessen KR Mirsky R. The repair Schwann cell and its function in regenerating nerves. J Physiol. (2016) 594(13):3521–31. 10.1113/JP270874 26864683 PMC4929314

[33] Teyssou E Vandenberghe N Moigneu C Boillee S Couratier P Meininger V. Genetic analysis of Ss18l1 in French amyotrophic lateral sclerosis. Neurobiol Aging. (2014) 35:1213 e9–e12. 10.1016/j.neurobiolaging.2013.11.023 24360741

[34] Pinto W Silva LHL Badia BML Yanagiura MT Souza PVS Oliveira ASB. Rapidly progressive bulbar-onset als due to Ss18l1 mutation. Rev Neurol. (2020) 176(3):217–9. 10.1016/j.neurol.2019.06.008 31522742

[35] Huttlin EL Bruckner RJ Paulo JA Cannon JR Ting L Baltier K. Architecture of the human interactome defines protein communities and disease networks. Nature. (2017) 545(7655):505–9. 10.1038/nature22366 28514442 PMC5531611

[36] Tando T Ishizaka A Watanabe H Ito T Iida S Haraguchi T. Requiem protein links Relb/P52 and the Brm-type Swi/Snf complex in a noncanonical Nf-kappab pathway. J Biol Chem. (2010) 285(29):21951–60. 10.1074/jbc.M109.087783 20460684 PMC2903353

[37] Michel BC D'Avino AR Cassel SH Mashtalir N McKenzie ZM McBride MJ. A Non-Canonical Swi/Snf complex is a synthetic lethal target in cancers driven by Baf complex perturbation. Nat Cell Biol. (2018) 20(12):1410–20. 10.1038/s41556-018-0221-1 30397315 PMC6698386

[38] Zhang F Boothby M T. Helper type 1-specific Brg1 recruitment and remodeling of nucleosomes positioned at the Ifn-gamma promoter are Stat4 dependent. J Exp Med. (2006) 203(6):1493–505. 10.1084/jem.20060066 16717115 PMC2118309

[39] Weider M Kuspert M Bischof M Vogl MR Hornig J Loy K. Chromatin-remodeling factor Brg1 is required for Schwann cell differentiation and myelination. Dev Cell. (2012) 23(1):193–201. 10.1016/j.devcel.2012.05.017 22814607

[40] Bai J Mei PJ Liu H Li C Li W Wu YP. Brg1 expression is increased in human glioma and controls glioma cell proliferation, migration and invasion *in vitro*. J Cancer Res Clin Oncol. (2012) 138(6):991–8. 10.1007/s00432-012-1172-8 22362300 PMC11824656

