# Corrigendum: Transcription factor SS18L1 regulates the proliferation, migration and differentiation of Schwann cells in peripheral nerve injury

**DOI:** 10.3389/fvets.2023.1324763

**Published:** 2023-11-03

**Authors:** Tianmei Qian, Pingping Qiao, Yingnan Lu, Hongkui Wang

**Affiliations:** ^1^Suzhou Medical College of Soochow University, Suzhou, China; ^2^Key Laboratory of Neuroregeneration of Jiangsu and Ministry of Education, Co-Innovation Center of Neuroregeneration, NMPA Key Laboratory for Research and Evaluation of Tissue Engineering Technology Products, Nantong University, Nantong, China; ^3^School of Overseas Education, Changzhou University, Changzhou, China

**Keywords:** transcription factor, SS18L1, Schwann cell, proliferation, migration, differentiation, peripheral nerve injury

In the published article, there was an error in affiliation [1]. Instead of “School of Biology and Basic Medical Sciences, Suzhou Medical College of Soochow University, Suzhou, China”, it should be “Suzhou Medical College of Soochow University, Suzhou, China”.

The authors apologize for this error and state that this does not change the scientific conclusions of the article in any way. The original article has been updated.

